# Enhancing the potential of children’s agency to achieve global sustainability

**DOI:** 10.1007/s13280-025-02134-8

**Published:** 2025-02-19

**Authors:** Tammy L. Elwell, Laura Nahuelhual, Marta Silva Fernández, Julie A. Bianchini, Steven D. Gaines

**Affiliations:** 1 Centro de Investigación en Dinámica de Ecosistemas Marinos de Altas Latitudes (IDEAL), Facultad de Ciencias, Universidad Austral de Chile, Valdivia, Chile; 2 Marine Science Institute, University of California at Santa Barbara, Santa Barbara, CA 93106-6150 USA; 3 Department of Social Sciences, Universidad de los Lagos, Osorno, Chile; 4 Instituto Milenio en Socio-Ecología Costera (SECOS), Santiago, Chile; 5 Fundación Bariloche, San Carlos de Bariloche, Argentina; 6 Instituto de Ciencias de la Educación, Facultad de Filosofía y Humanidades, Universidad Austral de Chile, Valdivia, Chile; 7 Millennium Nucleus to Improve the Mental Health of Adolescents and Youths, Imhay, Valdivia, Chile; 8 Department of Education, University of California at Santa Barbara, Santa Barbara, CA 93106 USA; 9 Bren School of Environmental Science and Management, University of California at Santa Barbara, Santa Barbara, CA 93106 USA

**Keywords:** Biosphere stewardship, Broadening participation in sustainability, Children’s connection to nature, Environmental education, Governance, Youth agency

## Abstract

Despite progress toward sustainability, it remains uncertain how we as a global society will make transformations needed at scale. We argue that intentionally involving children in sustainability efforts may catalyze promising changes in how we approach human-environmental crises. This involvement, however, entails much more than the traditional focus on exposing children to environmental education or sustainability learning. Here, we present a pathway where children move through incremental yet fluid stages, from rich experiences in nature in early childhood, to reflective activities, leadership development, and formal opportunities in sustainability governance in later childhood and adolescence. Each stage requires overcoming distinct barriers that vary across and within countries: we identify and discuss these main barriers, and suggest potential ways the sustainability sciences community can help to reduce them. We offer this proposed pathway as a first step toward ensuring young people’s involvement, agency, and stewardship in achieving global sustainability.

“*And if a few children can get headlines from all over the world just by not going to school, then imagine what we can all do together if we really wanted to.*”

Greta Thunberg at COP24 in Poland, December 2018.

## Introduction

Richard Louv’s 2005 book *Last Child in the Woods* spurred a palpable shift in the priorities of industrialized societies: children need opportunities to connect with nature. An emerging child-nature movement has begun to address this need, with diverse community groups leading efforts to make nature accessible to children and their families, especially in urban areas where safe natural spaces are scarce or nonexistent. Even in less industrialized regions and rural areas, where children whose families’ livelihoods and ways of life often depend more directly on ecosystems, such initiatives have reinforced the importance of time in nature—off screens or devices and engaged with the surrounding environment. These efforts have emerged in tandem with studies that have made apparent just how transformative nature can be for human wellbeing (see, for example, Soga and Gaston 2022). For children, these benefits may be even more pronounced. Positive nature experiences, early on, support myriad aspects of wellbeing, from overall development and learning (Chawla 2015; Kuo et al. 2022), to heightened creativity (Fjørtoft and Sageie 2000; Dowdell et al. 2011), to pro-environmental attitudes and behaviors (Wells and Lekies 2006; Rosa et al. 2018).

The benefits associated with time in nature have the potential to ripple out from children to families, communities and our global society and, if further guided and built upon, to transform our ability to achieve sustainability. Yet, even if it is reasonable to imagine these possibilities, it remains unclear how our global society might enhance children’s potential to obtain better, more equitable and more sustainable outcomes. In other words, here, we ask: how might early, positive experiences in nature be best leveraged to enhance youth’s potential to act as biosphere stewards?

We argue that ensuring children’s access to the natural world must be viewed as but one step in a larger concerted effort to enable youth to actively improve the world they live in. We envision this effort as a pathway where children progress through distinct stages intended to support and strengthen their potential as present-day actors in the sustainability movement who can wield agency and act collectively as biosphere stewards. We refer to agency in its broadest sense, which encompasses the idea that individuals and groups have the power to shape their own identities, exercise their rights, and pursue their goals within the opportunities and constraints presented by their social, cultural, and political contexts (Emirbayer and Mische 1998; Brown and Westaway 2011). Our intent, then, is to propose a pathway that helps to develop and channel the agency that children inherently possess.

We base this Perspective on our ongoing research with youth in rural and urban coastal regions of Chile, an upper-middle-income country, and California, a state in the high-income United States of America (USA), both marked by complex, and often stark, social inequities. It is important to state from the outset that we are not suggesting that this pathway serve as a panacea to achieve a more just and sustainable world, but rather as a framework from which to set certain processes in motion. We first reflect on why it is that young people are still largely viewed as passive agents (i.e., future inheritors of the biosphere) and why their efforts toward sustainability remain marginalized. We then discuss each stage of our proposed pathway toward expanding children’s stewardship, including opportunities that can make it more likely that youth experience each stage, as well as barriers that prevent or discourage involvement. Lastly, we suggest ways in which the sustainability sciences community can increase the likelihood that this pathway takes hold and helps enhance the potential of children’s agency for broader impact.

## Children in sustainability: Everywhere but nowhere

Children, persons from birth through age 17 (UNICEF 1989), are at once present-day child citizens and future adult citizens (Uprichard 2008). Here, we use the terms children, young people, and youth synonymously according to the United Nations’ “definition of childhood”, which encompasses adolescence. Although the concept of sustainability brings to the forefront the wellbeing of children and future generations, it also connotes delayed agency (i.e., children inherit the world as adults leave it). In sustainability discourse and in practice, it is not yet explicit that children hold agency here and now. Children are generally viewed more as passive recipients of future projections rather than as current agents of change.

This framing of children as future inheritors, rather than present-day actors, reinforces our global society’s status quo movement along unsustainable social-ecological trajectories (Cumming et al. 2014). This is not to say that we should pass the burden for a sustainable, equitable future on to children. Although our proposed pathway is intended for children, these efforts need to be led and carried out by adults (i.e., sustainability scholars who build collaborative networks with educators, parents, and community figures, among others). Younger generations face unprecedented challenges related to global change. More than ever, young people across a vast range of realities and contexts need spaces, dialogue, and tools to help guide journeys toward personal and community health and wellbeing, all the while learning to cope with difficult emotions intertwined with social-ecological crises (Barker and Franklin 2017; Kelsey 2020; Jones et al. 2023). To begin this process, as Sobel (1996) argues, we must encourage children to fall in love with nature.

Our proposed pathway is also not intended to downplay the contributions of children who have already engaged in organized efforts toward sustainability (see, for example, UNFCCC 2013). On the contrary, these examples underscore the potentially transformative role of expanding children’s contributions through an intentional frame, perhaps ensuring that changes take place sooner (Rana et al. 2020). The youth-led movement for climate justice and action underscores the powerful role young people can play in tipping global society toward a more equitable and sustainable future. The movement for climate action draws from decades of environmental education, a distinct movement stemming from the 1970s that promotes learning about the environment as a means to motivate informed decisions toward sustainability. Both the climate action movement and environmental education have contributed to heightened public awareness of complex human-environmental crises *and* the need for capacity building to confront these crises (Barker and Franklin 2017). Yet, despite this progress, the examples that demonstrate children’s ability to wield agency in efforts that could enhance sustainability are limited: they often come from higher-income countries and do not yet benefit from a broader framework and infrastructure that addresses the personal and community health aspects of global change, while fostering continuity in leadership as young activists grow and take on other responsibilities.

## A proposed pathway toward enhanced children’s stewardship

**Fig. 1:**
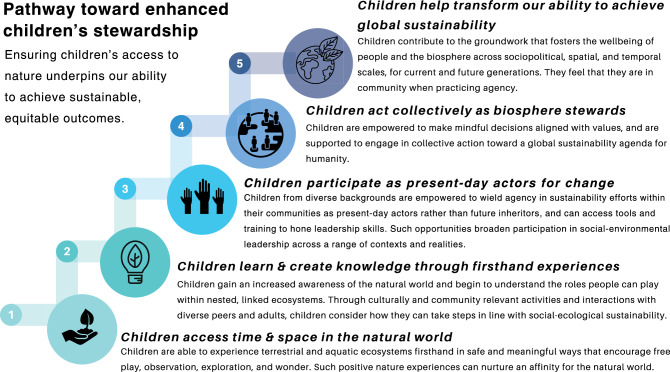
A proposed pathway for enhanced children’s stewardship, which creates conditions for young people to more formally join the ranks of global sustainability efforts. Each stage presents unique opportunities and barriers

Our proposed pathway toward enhanced children’s stewardship aims to build on and expand the existing efforts of young people like Vanessa Nakate, Yurshell Rodríquez Hooker, and Mitzi Jonelle Tan, who, from their respective regions and contexts, are leading shared agency toward a more just, sustainable world. Our proposal supports the notion of transformative education described in Sustainable Development Goal 4.7 of Agenda 2030 and aligns with UNESCO’s vision of education as a common good through which *all* children, as global citizens, may help realize a more sustainable and just world (UNESCO 2021). The proposed pathway involves five incremental stages, beginning with children’s direct engagement with the natural world and ending with young people’s enhanced ability to affect positive change toward sustainable, equitable outcomes (Fig. 1). We view these stages as malleable and fluid: stages may be adapted to individual and collective needs or realities, and youth can partake in aspects of previous stages to bolster the learning and growth intended at a later stage (e.g., continuously engage with the natural world, Stage 1, especially when taking on leadership).

**Fig. 2:**
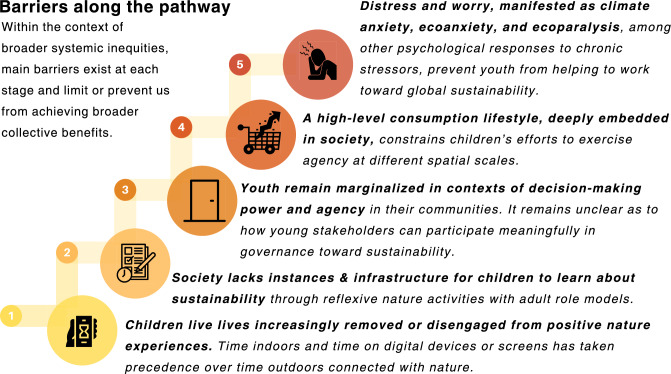
Main barriers that prevent or discourage involvement at each stage of the proposed pathway for enhanced children’s stewardship. These barriers are interconnected and can be found across the pathway ladder. However, for clarity, we identify and discuss main barriers at each stage. The color spectrum for the icons (from yellow, to orange, to red) was chosen for aesthetic reasons, so as to contrast with the color scheme used in Fig. 1

**Fig. 3:**
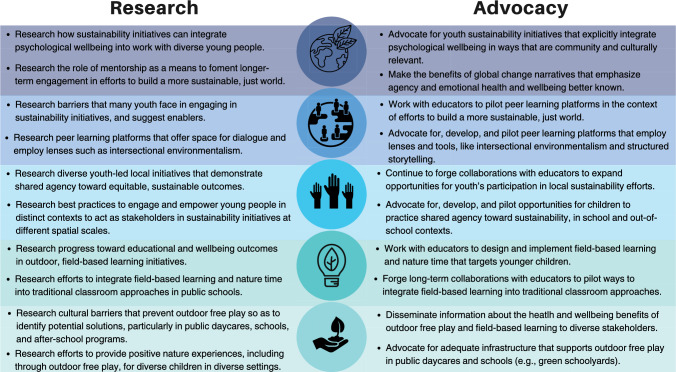
Ways in which the sustainability sciences community can help reduce barriers and increase the likelihood that young people experience stages of the proposed pathway toward enhanced biosphere stewardship (Fig. 1). We organize this call to action according to each stage of the pathway, as indicated by the icons carried over from Fig. 1. The research column suggests avenues of future research, while the advocacy column highlights opportunities for the community to engage in efforts toward broader societal impacts

Each stage in this process entails distinct barriers that vary across and within lower-, middle-, and higher-income countries. It is beyond the scope of this Perspective to delve into the nuances of context-dependent barriers that exist within broader systemic inequities (e.g., gender, racial, and socioeconomic inequities). In order to motivate work toward solutions, we consider the main barriers at each stage (Fig. 2) and suggest how the sustainability sciences community may contribute to breaking down these main barriers through both research and advocacy (Fig. 3). In so doing, we aim to spark creative collaboration and action in order to see this pathway begin to take hold in a broad range of contexts. This possibility entails more direct, ongoing engagement among sustainability scholars, educators, and policymakers, among others, and targets both formal (e.g., public schools and daycares) and informal educational settings (e.g., after-school programs).

### Stage 1: Children access time and space in the natural world

In this foundational stage, children are encouraged to explore the natural world in safe and meaningful ways, both at school and in informal contexts like after-school and community programs. The idea here is to maximize what Soga and Gaston (2022) describe as early nature experiences, which can forge a lifelong affinity for the natural world (Wells and Lekies 2006; Rosa et al. 2018). For example, positive nature interactions may encourage children to develop an empathetic perspective toward nature (Chawla 2020), a quality that can influence life trajectories. Conceptions of nature vary across places and cultures, change over time, and—particularly for children—largely depend on the sociocultural context in which they live (Adams and Savahl 2017; Sobko et al. 2018). For this Perspective, we adopt what Marris (2013) describes as a wider vision of nature that leads to an array of anthro- and eco-centric goals. This wider vision acknowledges different worldviews, while also reflecting inextricable relationships between people and nature.

Yet while the idea of what constitutes a positive nature experience is flexible, the timing of this stage is more straightforward. Research suggests that, overall, earlier is better (Chawla 2007). Such timing leverages what the unique life stage of early childhood offers: what psychologists call *lantern focus*. For younger children, nearly everything in the world is new and thus warrants wonder and awe (Gopnik 2009). Key emotive experiences—early on—can instill reverence for the natural world, and, in turn, foster stewardship (Chawla and Cushing 2007), which is defined as a response to sustainability challenges that encompasses care, knowledge, and agency (Peçanha Enqvist et al. 2018). Whether a child lives in a context where caretakers rely directly on ecosystems for material wellbeing (e.g., subsistence) or indirectly on ecosystems that support other aspects of wellbeing (e.g., recreation), that awe can nurture an affinity for nature.

A main barrier that hinders children from experiencing this foundational stage is the fact that children increasingly live lives removed or disengaged from positive nature experiences (see again Fig. 2). We categorize this barrier as cultural, since it encompasses a shift in societal and cultural preferences toward time indoors and on screens or devices (Singer et al. 2009; LeBlanc et al. 2015). Exceptions notwithstanding, this shift has occurred broadly, across the rural–urban spectrum, in lower-, middle-, and higher-income country contexts. Adult caretakers may prefer that children stay inside due to fear of illness or the inconvenience of getting dirty. Academics may be prioritized at the expense of outdoor play, even before grade school. This barrier also stems from socioeconomic inequities, given that in many places around the world, it simply may not be feasible to create and maintain safe, clean spaces in which children can play (Adams and Savahl 2015). Particularly in these contexts, even if there are safe spaces in which to access the natural world, caretakers work to meet basic needs and cannot readily accompany children in outdoor play. It is thus crucial that networks in interaction with the child’s family offer positive nature experiences, including outdoor free play, in public daycares, schools, and after-school programs.

Efforts that begin to address this barrier are well underway within the child-nature movement. These efforts often take place in urban areas where people lead lives more removed from their encompassing ecosystems. In these contexts, actors with diverse goals (e.g., NGOs) lead initiatives in schools and informal settings to increase access to the outdoors and encourage children and families to spend time in nature. End goals for these positive nature interactions vary according to the aims of the actors leading the initiative. For example, educational initiatives may focus on sparking interest in science, whereas government-sponsored public health programs may focus on increased physical activity. Such efforts often focus on children and families who cannot (or may not know how to) readily access the natural world, or, for a range of reasons, do not feel safe or welcome in attempting to do so. For many such efforts, access is thus understood as the ability—as opposed to the right—to benefit from the natural world (Ribot and Peluso 2003).

In terms of research that examines this proposed first stage, studies have documented dimensions of access to nature that go beyond physical access based on proximity to attend to cultural and linguistic diversity, among other dimensions (Wang et al. 2015; Rigolon 2016). This attention to diversity has spurred efforts to make outdoor spaces more inclusive, especially through outreach to populations who have been excluded or marginalized. Yet, although significant traction has been gained, most efforts to ensure access to the natural world have taken place in higher-income contexts. Research that identifies ways to provide opportunities for positive nature experiences for diverse children in diverse contexts is sorely needed. In tandem, general advocacy for outdoor free play in public educational settings (e.g., ensuring green schoolyards) may help make the benefits associated with positive nature experiences better known among broader audiences.

### Stage 2: Children learn and create knowledge through firsthand experiences

In this stage, children actively reflect on what they observe and experience in the natural world. Children continue to access space outdoors (i.e., outdoor free play), yet do so with diverse adult role models who encourage dialogue and questions and who show regard for the natural world and ways to learn from it. Thus, although this stage includes individual learning and development, collaboration takes precedence. In short, during this stage, children are engaged in field-based learning that builds reverence for the biosphere.

This curiosity-guided, field-based stage enables children to collectively build both local and broader awareness, while considering the potential roles people can play within nested, linked ecosystems. This may be accomplished through storytelling where children take on the perspectives of other species, through conducting inquiries and experiments, or by posing “why” questions that lend to learning about the history of a place (e.g., *Why does this plant grow here?*). The idea is that children develop multiple ways of knowing nature, from varied sources of knowledge. Recognizing multiple conceptions of nature *and* knowledge has been shown to build more inclusive learning communities, at least in the context of rural and urban Indigenous communities engaged in science education in the USA (Bang and Medin 2010). Such opportunities can employ inquiry-based learning platforms that are culturally and community relevant, such as the Learning in Places Collaborative in the USA (Tzou et al. 2021) and the Children’s Land (TiNi) Methodology (Box 1) in Peru. These initiatives employ learning in outdoor places as a strategy to prepare children to think and act as agents of change for the wellbeing of themselves, others, and nature. Place-based learning can plant seeds for shared agency (Bang and Medin 2010). Ideas for potential action may come forth from children, based on what they observe in their communities (Percy-Smith and Burns 2013).

We consider the main barrier at this second stage to be a lack of physical and programming infrastructure that supports learning in outdoor places in schools and after-school programs. This barrier is at once institutional, cultural, and economic. This barrier is institutional in that, overall, schools continue to follow a silo-style disciplines approach within the classroom, as opposed to integrated learning with real-world aspects (White and Delaney 2021). This barrier is also cultural in that society, as explained in our discussion of Stage 1, has shifted toward lifestyles spent indoors and on screens or devices (Singer et al. 2009; LeBlanc et al. 2015). Finally, this barrier entails economic constraints: field-based learning requires longer-term project funding and trained educator teams, as well as space and facilities that support positive nature experiences.

Traditionally, at least in the USA, field-based learning has targeted middle or high school students (James and Williams 2017) and has focused on the sciences. We argue that there is a pressing need to pilot ways for preschool and elementary school children to engage in this proposed second stage. Notably, initiatives may encompass social-ecological justice frames attuned to local communities and cultures (Tzou et al. 2021). Thus, children not only gain environmental or science literacy, but also learn about resource sustainability and conservation within their local context (Mongar 2023). Such initiatives would benefit from empirical research, in a range of contexts, to define and assess progress toward educational and wellbeing outcomes. Additionally, more work could be done to integrate field-based learning with traditional classroom learning, so as to suggest more feasible pathways forward for public schools and after-school programs with limited resources or where climate and cultural factors make outdoor learning difficult. Such research would go hand in hand with advocacy. For example, sustainability scholars could extend and deepen collaborations with educators to design and implement field-based learning initiatives for children.

Box 1: Field-based learning in the Children’s Land (TiNi) methodology The Children’s Land (TiNi) Methodology, which originated in Peru, exemplifies the type of field-based collaborative learning in the second stage of our proposal to enhance children’s stewardship. In TiNi, which has been implemented in more than 10 countries, particularly in Latin America, Mother Nature is recognized as and serves as a teacher alongside trained adults who guide children in developing empathy for life on Earth: the ability to feel, think, and act for the well-being of oneself, others, and the environment. TiNi can be carried out in small spaces (e.g., half a square meter of land) and in diverse socioeconomic and cultural contexts across the rural–urban spectrum. In this space granted by adults, children bond with nature, learn that they are part of her, and take actions that allow them to thrive by regenerating life and human relationships (ANIA 2023; Leguía Orezzoli 2023).

**Figure:**
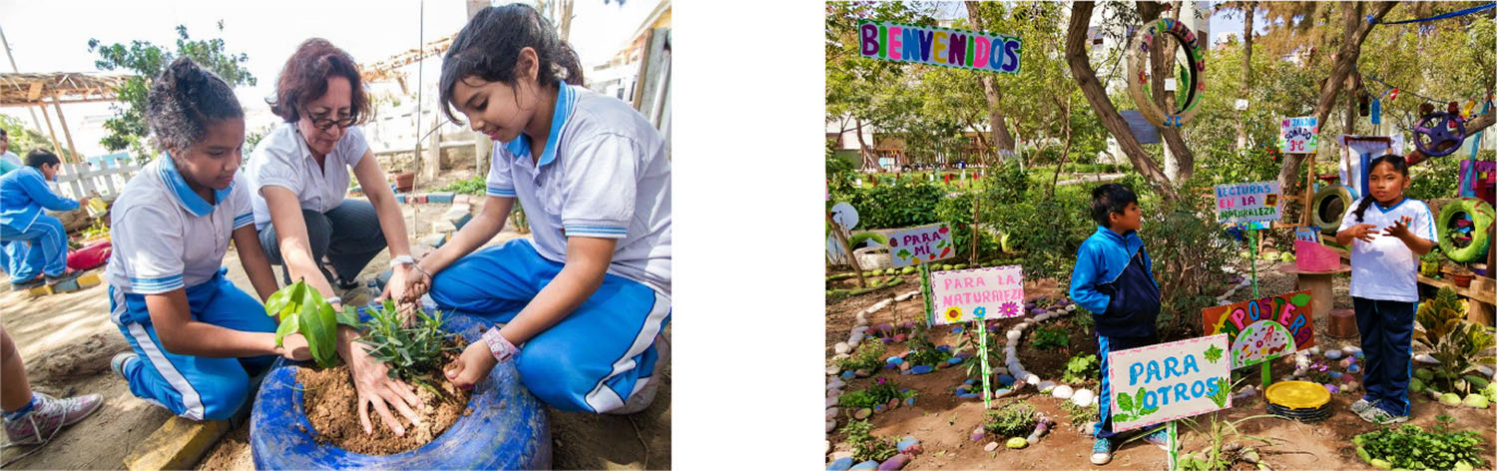
Photo credit: Asociación para la Niñez y su Ambiente

### Stage 3: Children participate as present-day actors for change

Our proposed third stage involves a more pronounced shift, where children move from playing and learning in the natural world to wielding agency in sustainability efforts within their communities. Here, children lead and contribute to what Lemos and Agrawal (2006) describe as interventions aimed to change environment-related knowledge and behaviors. Such culturally and community relevant interventions include actions and choices that lead to sustainability (Agrawal et al. 2022), such as working to ensure schools compost food waste or opt out of single-use plastic. Participatory processes related to environmental planning and policy could become more inclusive to children, allowing young citizens to partake in forums that seek public input (Hart 2013). Many children are already wielding such agency. However, as we have argued, these occurrences do not yet include broader efforts to help all children develop as social-environmental leaders in their communities, particularly in historically marginalized communities and in lower- and middle-income regions and countries. Such opportunities, both in schools and in community programs, would provide leadership development and mentorship, and emphasize shared agency in a local context. Throughout work in this stage, it is important to remind children to continue to make use of the health and wellness benefits from time in nature (Stage 1), in order to rest and maintain perspective.

A main barrier at this stage is that children remain largely marginalized in contexts of decision-making power and agency within their communities, despite empirical evidence that supports the benefits of children’s participation in affecting change (Freeman and Aitken-Rose 2005; Yunita et al. 2018). This stems from a lack of clear vision as to how children could participate meaningfully in formal interventions at different spatial scales (Percy-Smith 2010). Adults may view younger participants as too immature, or argue that youth ought to focus on studies or on helping their families rather than acting collectively toward sustainability. This barrier also encompasses rules for participation, including guardians’ consent and restrictions intended to protect children. In this regard, schools and youth-oriented community programs offer more feasible spaces from which children can work to change their immediate surroundings (e.g., school waste reduction and management, school or community gardens).

Efforts that begin to address this barrier stem from the sustainability movement and have focused on facilitating children’s participation as actors for change in school and community programs. With guidance, children can help inform the design of schoolyard and park infrastructure (Derr et al. 2013) and identify community relevant issues of environmental justice (Birmingham et al. 2017; Tzou et al. 2021). In natural resource-dependent communities, youth can wield limited agency in governance by practicing how to collectively manage local fisheries and forests that support livelihoods and ways of life (Zurba and Trimble 2014; Espinoza-Tenorio et al. 2022). Such examples speak to a growing recognition that children can participate in meaningful, consistent ways to improve their local surroundings.

More work is needed to broaden opportunities for children to practice shared agency toward sustainability in their communities (Chawla 2020). For example, a review of research that assesses diverse youth-led local initiatives could help identify best practices to engage and empower young people in distinct contexts. Likewise, through advocacy, sustainability scholars could leverage collaborations with educators to develop and pilot opportunities for children to practice shared agency starting in and then moving beyond their schools.

### Stage 4: Children act collectively as biosphere stewards

In this stage, the spatial scale of shared agency expands. Young leaders, empowered to act as present-day actors for change in their schools and communities, are supported to engage in collective action toward a global sustainability agenda for humanity. Through intentionally designed learning platforms in schools and informal contexts, such as community programs, young leaders dialogue with each other, share experiences, and collaborate across contexts. Leadership stays rooted in local work yet takes on an increasingly global perspective. Networks of young people identify as biosphere stewards who recognize environmental and human wellbeing as intertwined, and apply critical reflection to make decisions aligned with values. These stewards work to redefine “human development and progress” (Folke et al. 2011, p. 719) in ways that respect the biosphere and its capacity as a system that integrates all living things and their relationships (Steffen et al. 2011).

We see the greatest barrier at this stage as consumption lifestyles and habits entrenched in global economic growth paradigms and social conventions (Hirth et al. 2023). This barrier is institutional in that dominant political, economic, and social orders uphold a high-level consumption model for progress (Hirth et al. 2023). It is also cultural in that, especially in wealthier countries, consumption lifestyles have become normalized as habitual behavior (Hobson 2003; Gifford 2011). Although the moral implications of these social norms are increasingly being questioned (Otto et al. 2020), their behavioral momentum means change is difficult (Gifford 2011). The formidable scale of this barrier constrains youth’s ability to exercise agency. For example, mobilized stewards may attempt to aggregate actions at broader scales (e.g., work to see that all schools in a country or region opt out of single-use plastic), yet find that deeply entrenched conventions make it extremely difficult to do so. This can, of course, drive collective action to create change; yet, the degree of change needed can easily overwhelm.

Increasingly, young leaders have drawn attention to the roles children can play in shared agency (O’Brien et al. 2018). The movement for climate action and justice, including youth-led Fridays for Future that started from Greta Thunberg’s protest in Sweden, has underscored the potential of youth’s agency. In the ongoing Fridays for Future movement, young leaders have leveraged online communication platforms to dialogue with peers in and out of their own countries, thereby placing their personal experience in contact with a global setting. In effect, youth from diverse realities and contexts have galvanized around demands for intergenerational justice and a future desired, not feared (Foran et al. 2017). In this regard, intersectional environmentalism, defined as “an inclusive version of environmentalism that advocates for both the protection of people and the planet”, (intersectionalenvironmentalist.com; Thomas 2022) offers a relevant and promising framework from which to redefine human development and progress, where alternative growth paradigms and social conventions would enable sustainability (Hirth et al. 2023) that at once prioritizes and addresses social justice and equity. Within this vein, an emergent literature on climate education, mainly focused on college classrooms, discusses ways to create spaces for dialogue and to equip youth and educators with tools to navigate their journeys. For instance, structured storytelling activities can help young people identify and question dominant narratives, including those surrounding the climate crisis (Gray 2024). Structured storytelling can also help youth develop empathy and reach common ground, as in the case of Fiji’s traditional process of Talanoa Dialogue applied in climate-focused sustainability initiatives (Hautzinger 2024).

There is a pressing need for further research on peer learning platforms, particularly at the secondary school level and in diverse country contexts, in order to support the development of potential biosphere stewards (see, for example, UNFCCC 2013). Future research may usefully take into account the disproportionate barriers that youth in less privileged contexts face when acting collectively toward common goals, and suggest opportunities attuned to these realities. In terms of advocacy, the sustainability sciences community could work with educators to implement peer learning platforms with creative lenses that enable youth to reflect on their own journeys and work collectively to redefine human development and progress.

### Stage 5: Children help transform our ability to achieve global sustainability

The ultimate goal of the comprehensive approach we present here is to foster healthy, resilient social-ecological systems. The final stage of our proposed pathway reflects this aim. By this stage, groundwork is in place through which young people are better positioned to foster environmental and human wellbeing across spatial, temporal and sociopolitical scales, and feel that they are in community when practicing agency. This stage builds on the leadership development and mentoring in Stages 3 and 4, yet adds an explicit focus on equipping older youth leaders to share knowledge and build a sense of community with younger peers. As young people mature and assume increasing responsibilities as adults, they transition leadership roles to the next generation of children and youth. Mentorship plays a pivotal role in this process. As in the previous stages, these efforts would be carried out in both schools and beyond school (e.g., community programs) through diverse peer learning platforms.

Across these platforms, youth collaborate, support each other, and practice shared agency, while staying attuned to the needs of younger peers who may take on leadership as older peers move into other roles. Youth-led community gardens offer a prime example of platforms where adolescents and young adults can mentor younger peers in gardening and sustainability practices, and, in so doing, foster agency and community (Fulford and Thomson, 2013; as one example, see project in the United States in Box 2). Justice-centered learning initiatives in science education, in which youth take on social-ecological justice issues and do ‘science that matters’, offer another example of spaces where youth can practice collective action and share experiences and insights with younger peers (Birmingham et al. 2017).

Box 2: The power of mentorship in Acta Non Verba Youth Urban Farm Project Acta Non Verba (ANV) Youth Urban Farm Project in Oakland, California in the USA exemplifies the type of mentoring we envision in the fifth stage of our proposal to enhance children’s stewardship. In ANV’s youth-led gardens, which are located in historically marginalized communities where people struggle to meet basic needs, children learn to grow their own food alongside trained adults and youth leaders. This active web of mentors encourages children to respect themselves and respect the Earth, and builds trust and community. ANV implements a structured system where participants are encouraged to become Camp Leaders in Training, thereby increasing the likelihood that younger children will benefit from mentors from their own communities. Junior counselors, who are high school students and typically previous program participants, are paired with experienced counselors. Together, with guidance from adult mentors, youth counselors engage the younger children in organic gardening, cooking and eating harvested produce, and generally supporting ANV’s mission to “elevate life for youth … by challenging oppressive dynamics and environments through urban farming and access to the natural environment” (https://anvfarm.org/).

**Figure:**
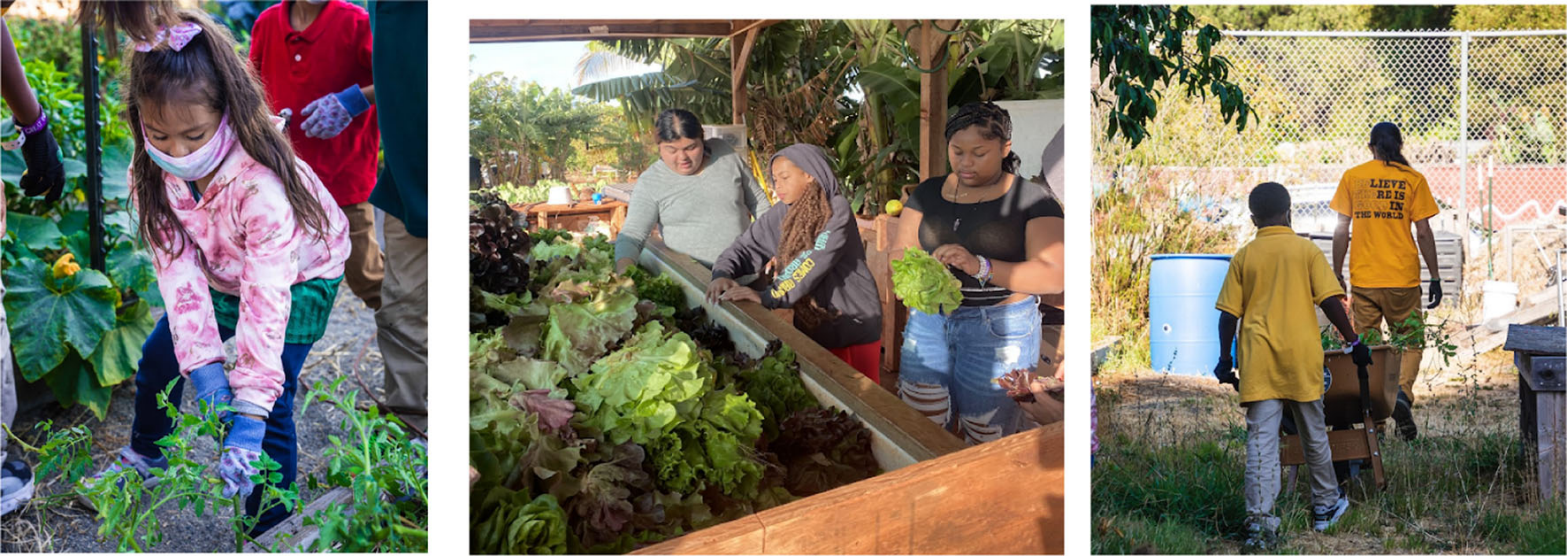
Photo credit: Acta Non Verba Youth Urban Farm Project

At this final stage, we view distress and worry, manifested as climate anxiety, ecoanxiety, and ecoparalysis, among other psychological responses to chronic stressors, as main barriers that prevent youth from transforming our ability to achieve global sustainability. These psychological responses are complex, nuanced subjects of emergent research on how youth experience uncertainty associated with social-ecological change (e.g., living on a warming planet). Although this research has focused more on youth in higher-income countries (see, for example, Ramadan et al. 2023), there is evidence that youth across a range of realities experience this distress. For example, youth in India and the Philippines, lower-middle-income countries, expressed high levels of worry and negative beliefs associated with both climate change *and* perceived inadequate government responses to climate change (Hickman et al. 2021).

Thus, just as the scale of change needed to achieve global sustainability can easily overwhelm (see again, main barrier in Stage 4, Section “Stage 4: Children act collectively as biosphere stewards”), portrayals of global crises—and experiencing detrimental global environmental change either firsthand or virtually—can result in feelings of worry and despair, particularly in youth (Burke et al. 2018). Several factors at different spatial scales influence how young people experience *climate anxiety*, which Crandon et al. (2022) suggest might be termed ‘climate distress’ or ‘climate empathy’ to more aptly capture this reasonable response that youth undergo when learning about climate change. The broader term *ecoanxiety*, defined as the chronic fear of environmental doom, encompasses feelings of helplessness, hopelessness, loss, grief, and frustration (Coffey et al. 2021). Critically, these responses can lead to inactivity and disengagement in sustainability efforts. That is, these strong feelings associated with a compromised planet can result in what many would call apathy or indifference, but what Albrecht (2011) suggests is better described as *ecoparalysis*, the inability to meaningfully respond to sustainability challenges.

These barriers are psychological in that responses can manifest as negative impacts on mental health and wellbeing, including anxiety and depression (Doherty and Clayton 2011). These barriers are also cultural in that they reflect a tendency to document tragedies without necessarily contributing to potential solutions (Lubchenco and Gaines 2019). And yet, science writers and scientists increasingly frame information and ideas about the state of the planet through lenses that emphasize agency, perhaps due to heightened awareness that doing otherwise can backfire (Johns and Jacquet 2018; Kelsey 2020). At the same time, in sustainability initiatives that engage children, there is an urgent need to make more well known the importance of emphasizing shared agency (Murray et al. 2023).

An emergent literature has begun to address the nexus of psychological wellbeing and biosphere health, and what these interactions imply for youth working toward a more sustainable and just world (Chawla 2020; Murray et al. 2023). A key theme in these works is the need to prioritize community-care and self-care. This intentional care includes fostering a sense of belonging through community development (Fulford and Thompson 2013), employing community healing processes to address strong emotions associated with injustices (Gonzaléz-Hidalgo et al. 2022), leveraging action framed within hope *and* grief (Jones et al. 2023), and regularly employing restorative practices like positive nature experiences, play, and rest (Chawla 2020). Indeed, helping youth to identify and channel strong feelings may help to spark a new kind of advocacy for the biosphere, where mental health and wellbeing act as guiding forces in efforts to build a more equitable, sustainable world (Lertzman 2015).

Future studies may examine how sustainability initiatives explicitly integrate psychological wellbeing components in work with young people. Again, these emerging initiatives tend to take place in higher-income countries (e.g., Murray et al. 2023); there is a pressing need for studies that pilot and assess sustainability-wellbeing initiatives with diverse children in more diverse contexts. Further, more research is needed on the role of mentorship as a means to support longer-term engagement in sustainability efforts. Few studies discuss peer mentorship among youth, despite the growing recognition that mutual support plays a key role in shared agency (Foran et al. 2017; Fine et al. 2023). Through advocacy, scholars could support peer learning platforms that integrate mentorship and explicitly address emotional and psychological wellbeing. Similarly, the sustainability sciences community can continue to stress the importance of global change narratives that emphasize shared agency along with personal and community wellbeing.

## Piloting this pathway in real-world settings

Given the challenges we face as a global society marked by vast disparities, some might dismiss this proposed pathway as unfeasible. However, we emphasize that aspects from each of the proposed five stages are well underway (see again Fig. 1). In order to apply the proposed path in real-world settings, in addition to the avenues of future research and advocacy specified in Fig. 3, we suggest pilot schools that integrate the pathway’s proposed stages into their curriculum and pedagogy. To assess impact, a comparison could be drawn from before and after the pilot school intervention, or a quasi-experimental research design could be employed with two groups within the same pilot school. Such a piloting process would unfold over several years to allow for long-term evaluation of key indicators, both overall and at each of the proposed stages. As examples, overall enhanced agency could be assessed through metrics of initiatives and autonomy (e.g., number and kind of initiatives proposed by the students themselves; students’ capacity to identify, approach, and address sustainability challenges in their communities), and the impact of positive nature experiences in Stage 1, for instance, could be assessed using a culturally relevant connectedness to nature scale (see, for example, Sobko et al. 2018). Educators and school leadership, along with collaborators from the sustainability sciences community, would engage in capacity strengthening and specialized training to support students, their families, and members from the broader community in these endeavors.

## Conclusion

Here, then, we contribute a novel, comprehensive pathway that frames children’s formal involvement in a global sustainability agenda (see again Fig. 1). We argue that the proposed pathway would help more young people contribute to transformative change toward a more sustainable and just world, in ways that ensure generational continuity and prioritize psychological health and wellbeing. We emphasize that the work in each stage of the pathway entails what Atkinson and Ray (2024, p.14) describe as the “slow work of cultivating relationships and equitable processes” toward the goal of engaging a broader cohort of potential biosphere stewards.

It is in this spirit—in acknowledging the slow work that this pathway entails—that we have identified and discussed main barriers at each stage that prevent or discourage young people from more formally joining the ranks of sustainability efforts (see again Fig. 2). Understanding and addressing these barriers is a first step toward ensuring young people’s involvement, agency, and stewardship—processes that we have framed through the incremental stages of our proposed pathway.

The sustainability sciences community, with increasingly diverse cross-disciplinary initiatives that span geographic regions and engage a range of participants, is well positioned to help break down these barriers and increase the likelihood that this pathway takes hold. We have suggested pilot schools as one means to test, adjust, and scale up our proposed path, and, for each stage of the proposed pathway, we have suggested specific actions within two broad approaches: research and advocacy (see again Fig. 3). Although each stage lends itself to its own focused agenda attuned to context, perhaps most apparent is both the need and promising opportunity for the sustainability sciences community to forge and deepen collaborations with educators, particularly in public schools, daycares, and after-school programs, in order to help children make a difference in realizing a more just and sustainable world.

## Data Availability

No new data were created or analyzed during this study. Data sharing is not applicable to this article.
